# Carbon ion triggered immunogenic necroptosis of nasopharyngeal carcinoma cells involving necroptotic inhibitor BCL-x

**DOI:** 10.7150/jca.46316

**Published:** 2021-01-01

**Authors:** Cihang Bao, Yun Sun, Bilikere Dwarakanath, Yuanli Dong, Yangle Huang, Xiaodong Wu, Chandan Guha, Lin Kong, Jiade J. Lu

**Affiliations:** 1 Department of Radiation Oncology, Shanghai Proton and Heavy Ion Center, Fudan University Cancer Hospital, Shanghai, China; 2 Department of Research and Development, Shanghai Proton and Heavy Ion Center, Shanghai, China; 3 Shanghai Engineering Research Center of Proton and Heavy Ion Radiation Therapy, Shanghai, China; 4 Department of Radiation Oncology, Fudan University Shanghai Cancer Center, Shanghai, China; 5 Department of Radiation Oncology, Albert Einstein College of Medicine, Montefiore Medical Center, Bronx, New York, USA

**Keywords:** carbon ion irradiation, necroptosis, immunogenic cell death, nasopharyngeal carcinoma, photon-resistant cells, mixed lineage kinase domain-like pseudokinase

## Abstract

To explore the potential and mechanisms of necroptosis, a form of immunogenic cell death, induced by carbon ion as compared to photon beams in established photon resistant- (PR-) and sensitive nasopharyngeal carcinoma (NPC) cells. MLKL is considered a central executor of necroptosis and phosphorylation of MLKL (p-MLKL) was a critical event of necroptosis. The clonogenic survival and DNA microarray demonstrated that after repeated photon irradiation, radiosensitive NPC cells became apoptosis-resistant but could be effectively inhibited by carbon ion irradiation. The relative biologic effectiveness (RBE) at D10 and D37 were 2.15 and 2.78 for PR-NPC cells. Carbon ion induced delayed DNA damage repair, cell cycle arrest, cytogenetic damage, morphological change and cell necrosis, indicating the possibility of necroptosis in both PR- and sensitive NPC cell types. The lower expression of necroptotic inhibitors (caspase-8 and Bcl-x) and higher level of MLKL in PR-NPC cells showed it was relatively more predisposed to necroptosis compared to the sensitive cells. Subsequent experiments demonstrated the significant upregulation of p-MLKL in the PR-NPC cells treated by carbon ion (4 Gy) compared with photon irradiation at both physical (4 Gy) and RBE (10 Gy) doses (P≤0.0001). Moreover, carbon ion induced a robust (up to 28 folds) p-MLKL in the PR-NPC cells as well as sensitive cells (up to 6-fold) coupled with a lower level of BCL-x expression and increased GM-CSF implicated in resculputure of immune system. These results suggested that carbon ion could induce necroptosis of NPC cells, especially in PR-NPC cells, and its mechanisms involve BCL-x.

## Introduction

The outcome of nasopharyngeal carcinoma (NPC) failed locally after high-dose radiotherapy (RT) is usually dismal, and the inherent resistance induced by the first course of RT may be one of the main reasons for treatment failure after salvage re-irradiation using photon beams 1, 2. Carbon ion (CI) beams have a superior dose distribution to the tumor volume and a higher relative biological effect (RBE) for cell killing compared to photon beam 3, and is a clinically proven salvage modality for locoregionally recurrent NPC (LR-NPC) after definitive photon based RT 4. Re-irradiation with CIRT provides promising survival rates with acceptable severe (grade 3 or 4) toxicity profile (mucosal necrosis: 9.3%, xerostomia: 1.3%, and temporal lobe necrosis: 1.3%) for LR-NPC patients 4. Furthermore, distant metastasis was less frequent in patients received salvage CIRT for local failure 5, 6. The 1 year overall survival (OS) and distant metastasis-free survival (DMFS) rates for the LR-NPC patients (mostly with stage rIII-IV disease) salvaged by CIRT reached 98.1% and 96.2%, respectively 4, substantially better than those rates (92% and 90.5%, respectively) of patients with better prognostic factors (only with 40.3% rIII-IV stages) salvaged by photon-based IMRT 1.

Carbon ion induced immunogenic cell death (ICD) may be one of the main reasons of the reduced distant metastasis and higher survival rates. Necroptosis, a programmable form of necrosis, has been reported as a novel response mode of cancer cells to ionizing radiation 7-9. Ablative hypofractionated radiation therapy at ≥10 Gy per fraction enhances killing of non-small cell lung cancer with high receptor interacting serine/threonine kinase (RIPK)-3 expression via preferential stimulation of necroptosis 9, as a caspase-independent form of ICD 10-12, which is induced in apoptosis-resistant cells 13, 14 and involves the activation of RIPK1, RIPK3, and the pseudokinase mixed lineage kinase like (MLKL) 14, 15. MLKL is considered a central executor of necroptosis and its phosphorylation was a critical event of necroptosis 16. The necrosome complex consisting of RIPK1 and RIPK3 phophorylates MLKL leading to its oligomerization and translocation to the plasma membrane 15. This ruptures the membrane resulting in inflammatory response through the release of damage associated molecular patterns (DAMPs) and cytokines 11, 14, 15. However, induction of necroptosis in human tumors, including NPC, after carbon ion irradiation has yet to be studied.

The aim of the present study was to investigate the potential and the mechanisms of necroptosis induced by carbon ion beams as compared to photon beams in established photon-resistant NPC (PR-NPC) cells and the relative photon-sensitive parental cells. The involved regulator proteins, cytokine and chemokine were also investigated. This study will give an implication of the unique biological effects of carbon ion on NPC cells and show the potential of carbon ion inducing immunogenic necroptotic cell death and thus the potential to initiate a radio-immunogenic effects, which paves the way of carbon ion for further application in radio-immunotherapy.

## Material and methods

### Cell culture and establishment of X-ray resistant cells

An NPC cell line CNE-2 (a gift from the Center for Molecular Medicine, Xiangya Hospital, Central South University, Changsha, China), a human liver carcinoma cell line HepG2 and a laryngeal cancer cell line Hep2 (both purchased from Shanghai Zhong Qiao Xin Zhou Biotechnology Co., Ltd, China) were cultured in RPMI-1640 supplemented with 10% fetal bovine serum in a 37°C with humidified atmosphere and 5% CO_2_. The CNE-2-RR cell line was established according to a method described previously 17. Both CNE-2 and CNE-2-RR cells were collected for STR genotyping, which was performed by Shanghai Biowing Applied Biotechnology Co. LTD, Shanghai, China and GENEWIZ, Inc. Suzhou (**Table S1**).

### Global gene expression by DNA microarray analysis

The procedure of DNA microarray analysis were described previously 18. We compared CNE-2 and CNE-2-RR cells once in our previous study (Group 2) 18 and here we conducted second independent experiments (Group 1) to confirm CNE-2-RR cells becoming an apoptosis-resistant cells. All samples were hybridized to Human U133 2.0 plus arrays (Affymetrix). We defined differential expression gene as genes with 2-fold changes of CNE-2-RR cells compared with CNE-2 cells. Comparison of multiple biological experiments was facilitated with Venn diagram (http://www.pangloss.com/seidel/Protocols/venn.cgi). KEGG pathway enrichment was performed and presented in pictures by using R (version 3.5.3).

### Irradiation and clonogenic survival assay

The procedures of radiation and clonogenic survival assay were described previously 19-21. Briefly, CIRT was delivered with a homogeneous spread-out Bragg peak with energy of 148.3~180.3 MeV/u (linear energy transfer, LET 315.7 keV/μm) on the target. A 225 kVp X-ray beam (PXi precision X-RAD 225, dose rate=3.198 Gy/min, 225kV, 13.3mA, 40cm SSD) was used as the reference photon beam. Following irradiation, cells were harvested by trypsinization, counted and plated for a colony-forming assay and cultured for 7-14 days in RPMI-1640 supplemented with 10% FBS. RBE-10 and RBE-37 values were calculated as the ratio of the D_10_ and D_37_ of CIRT to those of 225kVp X-rays.

### Nuclear morphological characteristics

Vital dye (Hoechst 33342) was added to cell culture medium at 24 hours and 48 hours after irradiation, incubated at 37°C for 5 min, and observed under a fluorescence microscope (Olympus BX41). Frequency of cells with micronuclei, buds, bridges, mitotic catastrophe and apoptosis, were counted according to the criteria described earlier 22-26. At least 300-900 cells in each dish from randomly selected fields were counted.

### DNA damage analysis by γ-H2AX assay

The assay was performed according to the procedures described before 27. Briefly, both cells were fixed at 1 hours and 24 hours after irradiation using 4% paraformaldehyde and permeabilised using 0.5% (v/v) Triton-X/PBS for 10 min, before blocking with 5% (w/v) goat serum (Beyotime Biotechnology, C0265, China) and 0.1%Tween20 in PBS for 30 min. Cells were stained with anti-phospho-Histone H2A.X(Ser139)(20E3) Rabbit mAb (1:200; Cell Signaling Technology, CST 9718S, USA) for 2 hours at room temperature, then washed and incubated in AlexaFluor 488 conjugated affinipure donkey anti-rabbit IgG (H+L) antibody (1:400, Jackson ImmunoReasearch Laboratories, USA) for 1 hours at room temperature. Cells were washed and counterstained with DAPI (Beyotime Biotechnology, C1005, China) and images were acquired with Nikon Eclipse Niu microscope and Evolve 512 Photometrics EMCCD using 20 × objective.

### Cell cycle analysis

Cell cycle distribution was analyzed at 24 hours and 48 hours after irradiation by flow cytometry 17. Briefly, Cells were fixed with 70% ethanol in PBS, stored at -20°C overnight and stained with PI/RNase staining buffer (BD Pharmingen, San Diego, CA, USA) according to the manufacturer's protocol.

### Apoptosis and necrosis analysis

Externalization of phosphotidylserine was assessed using FITC-Annexin V Apoptosis Detection Kit (BD Pharmingen, San Diego, CA, USA) according to the protocol provided by the manufacturer. Briefly, cells were trypsinized and washed twice with cold PBS (250g, 5min), and re-suspended in 100µl binding buffer before incubating with 5µl of FITC-Annexin V for 15min at room temperature in the dark. Another 400µl binding buffer was added and incubated with 5µl of PI for 30 seconds and cells were analyzed on a Guava easyCyte HT (Millipore, Billerica, MA, USA). A minimum of 10,000 cells were analyzed in each sample.

### Western blotting

Twenty microgram of whole cell lysate were separated by 7.5% SDS-PAGE (Bio-Rad) and transferred to PVDF membranes (Bio-Rad). Membranes were blocked for 10 min, followed by incubation with rabbit polyclonal antibodies overnight at 4°C. The membranes were then washed three times and incubated with horseradish peroxidase (HRP)-conjugated secondary antibody (1:3000; CST 7074S) for 1 h. After three final washes, the blots were visualized using an ECL detection system (Bio-Rad). Primary antibodies and concentrations used are indicated as follows: anti-Caspase-8 (1:1000; CST 4790S), anti-MLKL (1:1000; CST 14993S), anti-phospho-MLKL (1:1000; CST 91689S), anti-BCL-x (0.3 µg/mL; R&D Systems AF800), anti-PARP (1:1000; CST 9542S), anti-RIP (1:1000; CST 3493S), anti-RIP3 (E1Z1D) (1:1000; CST 13526), anti-GAPDH (1:1000; CST 5174S) and anti-COX IV (1:1000; CST 4844S).

### Protein arrays

Cell lysates obtained at 48 hours after irradiation (100 μg protein), were incubated with an array of antibodies against human apoptosis related proteins (ARY009; R&D Systems) and then were processed as per the manufacturer's protocol. The films were scanned with transmission-mode scanner and pixel densities analyzed with ImageQuant (GE Healthcare, Chicago, IL, USA).

### Multiplexed cytokine assay

The spent culture media were collected 48 h after irradiation and centrifuged for 30 min at 1500 r.p.m. to remove cell debris and stored at -80°C. Multicytokine analyses were performed using Luminex technology using the Milliplex map kit (HCYTOMAG-60K; 2962164, Merck Millipore Darmstadt, Germany) containing magnetic beads for quantification of 41 human cytokines and chemokines. Assays were performed according to the manufacturer's instructions. Briefly, after plates were pre-wet, allow the culture supernatant to centrifugate for 10 minutes at 10000rpm. Added 25 μl standard or control to appropriate wells and 25μl assay buffer to background and sample wells, then added 25 μl appropriate Matrix Solution to background, standards, and control wells. Culture supernatant (25 μl) were diluted 1:1 with assay buffer and added to the plate in sample wells. 25 μl of precombined beads was added, then incubated for 16-18 hours on a plate shaker at 700 r.p.m at 4°C temperature. Plates were washed twice with wash buffer and incubated 25μl of detection antibody for one hour on a plate shaker. 25μl of strepatavidin-PE conjugate was added to each well, and the plate was shaken at 600 rpm for 30 minutes at room temperature. Finally, plates were washed two times with wash buffer and 150 μl of sheath fluid were added to each well. Plates were read using a Luminex 200™ (Luminex, Austin, USA). Data was analyzed sing MILLIPLEX Analyst.V5.1 as per the instructions provided. A standard curve for each cytokine was generated by mixing known concentrations of recombinant human cytokines.

### Data analysis of pan-cancer samples using cBioPortal and STRING database

Gene expression data from cancer available at the TCGA database (TCGA, PanCancer Atlas) was assessed using the cBioPortal (http://cbioportal.org) to investigate the tendency of co-occurrence of genomic alterations 28 of MLKL and BCL2L1 (also known as BCL-x) in pan-cancer cohorts (32 studies, 10967 patients). We then used the STRING database 29 to predict protein-protein association.

### Statistical Analysis

We evaluated the statistical significance using two way ANOVA by the GraphPad Prism version 7.00 for Windows (GraphPad Software, San Diego California USA, www.graphpad.com). The significance values were denoted as '*'(0.01<p≤0.05), '**' (0.001<p≤0.01), '***' (p≤0.001) and '****' (p≤0.0001). Each experiment was repeated at least twice unless otherwise specified.

## Results

### Carbon ion radiation inhibited photon resistant human NPC cell lines

**Figure 1:**
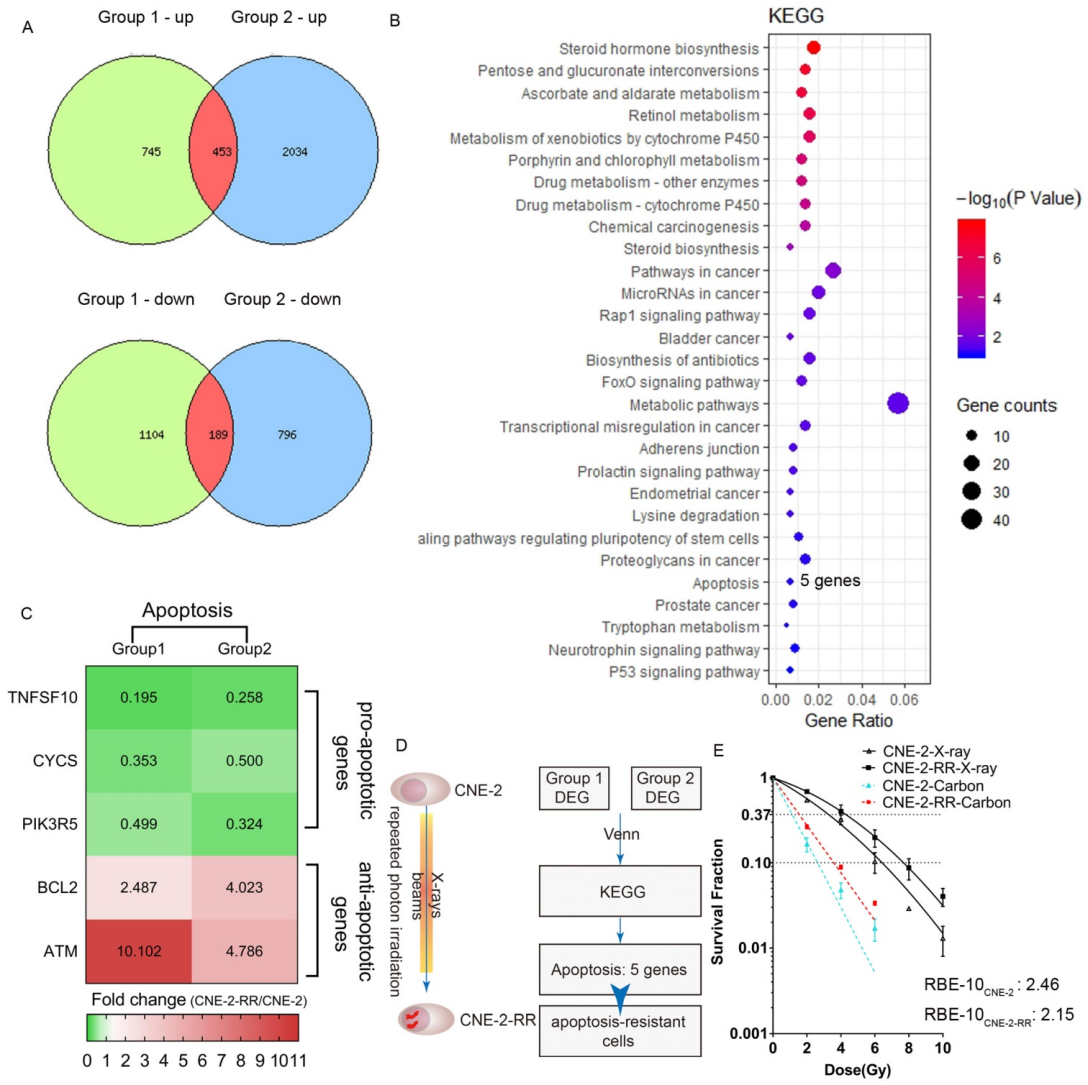
** Carbon ion radiation inhibited photon resistant human NPC cell lines.** (A) We did two independent experiments (Group 1 and Group2) of DNA microarray analysis and defined differential expression gene (DEG) as genes with 2 folds changes of CNE-2-RR cells compared with CNE-2 cells. Comparison of multiple biological experiments was facilitated with Venn diagram (http://www.pangloss.com/seidel/Protocols/venn.cgi). (B) KEGG pathway enrichment was performed. We found that apoptosis pathway (including 5 genes: TNFSF10, CYCS, PIK3R5, BCL2, ATM) was one of the enrichment pathways affected following repeated irradiation and (C) pro-apoptotic genes (TNFSF10, CYCS, PIK3R5) were down-regulation and anti-apoptotic genes (BCL2, ATM) were up-regulation in CNE-2-RR cells. (D) These results suggested that after repeated photon irradiation, NPC cells would be an apoptosis-resistant cells (named CNE-2-RR cells). (E) Dose-response curves for clonogenic survival of NPC (CNE-2 and CNE-2-RR) cells after treatment of different dose of X-ray and carbon ion.

To compare the human CNE-2 and CNE-2-RR cells, we carried out global gene expression using microarray and conducted KEGG pathway enrichment to gain insight into alterations in the pathways indicated by changes in the gene expression after repeated photon irradiation on CNE-2 cells. We found that apoptosis pathway (including TNFSF10, CYCS, PIK3R5, BCL2, ATM genes) was one of the enrichment pathways affected following repeated irradiation and pro-apoptotic genes (TNFSF10, CYCS, PIK3R5) were down-regulation and anti-apoptotic genes (BCL2, ATM) were up-regulation in CNE-2-RR cells (**Fig.1A-D**). These results suggested that after repeated photon irradiation, NPC cells became apoptosis-resistant (named CNE-2-RR cells). We previously demonstrated the RBE at the 10% survival level (RBE-10) was 2.46 for CNE-2 cells compared with 1.95 for PR-NPC cells (CNE-2R, albeit with a total dose of 100 Gy), respectively 21. Consistently, the RBE-10 of our newly established PR-NPC cells (CNE-2-RR cells) was 2.15 (**Fig. 1E** and **Table 1**). These results indicated the effective inhibition effects of carbon ion to both CNE-2 cells and photon resistant CNE-2-RR cells.

### Carbon ion beam induces delayed DNA damage repair, cell cycle arrest, cytogenetic damage, morphological change and cell necrosis indicating the possibility of necroptosis

**Figure 2:**
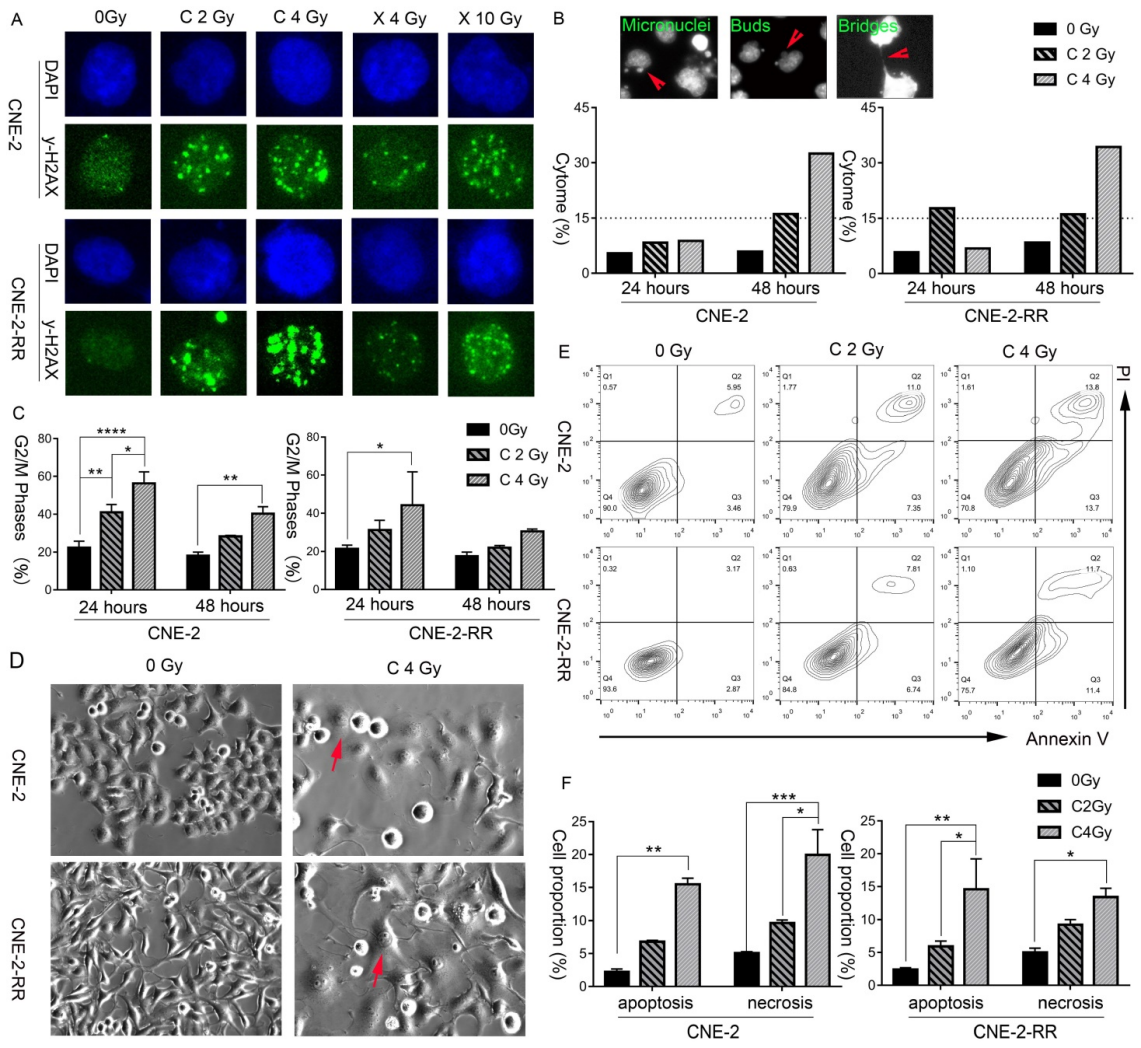
** Carbon ion radiation induced delayed DNA damage repair, cell cycle arrest, cytogenetic damage, morphological change and cell necrosis, indicating the possibility of necroptosis.** (A) CNE-2 and CNE-2-RR cells DNA damage repair at 24 hours following X-ray or carbon-ion exposure demonstrated by fluorescence imaging of γ-H2AX loci. Original magnification: 200 ×. (B) Fluorescence micrographs of Hoechst 33342 stained cells showing micronuclei, buds, bridges. And induced fraction (%) of cytogenetic damage (cytome) at 24 hours and 48 hours after carbon ion irradiation. Original magnification: 200 ×. (C) Percentage of cells in the G2/M (mean ± SEM) phases at 24 and 48 hours following exposure to carbon beams. *P≤0.05, **P≤0.01, ***P≤0.001, ****P≤0.0001. (D) Light microscopic images showing typical morphological features of necroptosis (flattened and enlarged cells: marked by arrows) in carbon irradiated cells. Original magnification: 200 ×. (E) Bivariate plots of Annexin-V and PI in CNE-2 and CNE-2-RR cells at 48 hours following carbon ion irradiation (Q2: necrosis; Q3: apoptosis). (F). Mean value of fractions of carbon ion-induced apoptotic (Annexin+ cells) and necrotic (including necroptosis) (PI+) cell death at 48 hours. *P≤0.05, **P≤0.01, ***P≤0.001, ****P≤0.0001.

The induction and repair of DNA damage, mainly the double strand breaks (DSB), are among the major contributing factors of radiation induced cell death. Thus, after exposure of different doses of carbon ion and X-ray beams, the γ-H2AX loci of CNE-2 and photon resistant CNE-2-RR cells were monitored by fluorescence microscopy at 1 hours and 24 hours, to demonstrate the DNA damage repair. At 1 hour after X-ray or carbon ion irradiation, both CNE-2 and CNE-2-RR cells start to show γ-H2AX fluorescence signals at a similar intensity, indicating carbon ion and X-ray induced DNA broken **(Fig. S1)**. At 24 hours after irradiation, carbon ion induced brighter and larger γ-H2AX fluorescence signals than that X-ray in both cells, suggesting that the carbon ion beams induced DSB broken was more difficult to repair than that induced by X-ray** (Fig. 2A).**

Moreover, a dose dependent cytogenetic damage at 48 hours following carbon ion irradiation was monitored, and was obviously higher than that at 24 hours (**Fig. 2B**).The cytogenetic damage at 48 hours is consistent with the partial release of radiation-induced G2 block at 48 hours (**Fig. 2C**), as micronuclei are expressed in post-mitotic cells. The cell cycle using flow cytometry at 48 hours after 2 Gy and 4 Gy CIRT suggested a dose dependent delay in the cell cycle, with a block in the G2+M phases of the cell cycle (**Fig. 2C**). These blocks were apparent in both cell types at 48 hours following 2 Gy and 4 Gy CIRT.

Furthermore, morphological features of these cells were further monitored by bright field microscopy. Representative photomicrographs (**Fig. 2D and Fig. S2**) clearly showed swollen cells with flattened morphology following irradiation, which was more prominent and frequent in the photon resistant (CNE-2-RR) cells irradiated with carbon at 48 hours. Fluorescence microscopy of the nuclear morphology also suggested a higher frequency of hypertrophy of the nucleus in carbon-ion irradiated cells, particularly in CNE-2-RR cells (**Fig. S2**).

Apoptosis and necrosis/necroptosis at 48 hours was then analyzed by flow cytometry using Annexin-V and PI staining (**Fig. 2E and 2F**). At 48 hours after 2 Gy CIRT, the necrosis/necroptosis rates of CNE-2 and CNE-2-RR cells were 9.64±0.65% and 9.24±1.54%, respectively. Those rates were 19.94±8.61% and 13.42±2.95% after 4 Gy CIRT, showing a dose dependent necrosis/necroptosis ratio. These results suggested that carbon ion might induce necrosis/necroptosis in NPC cells.

### Carbon ion irradiation induced phosphorylation of the critical necroptosis marker protein MLKL

**Figure 3:**
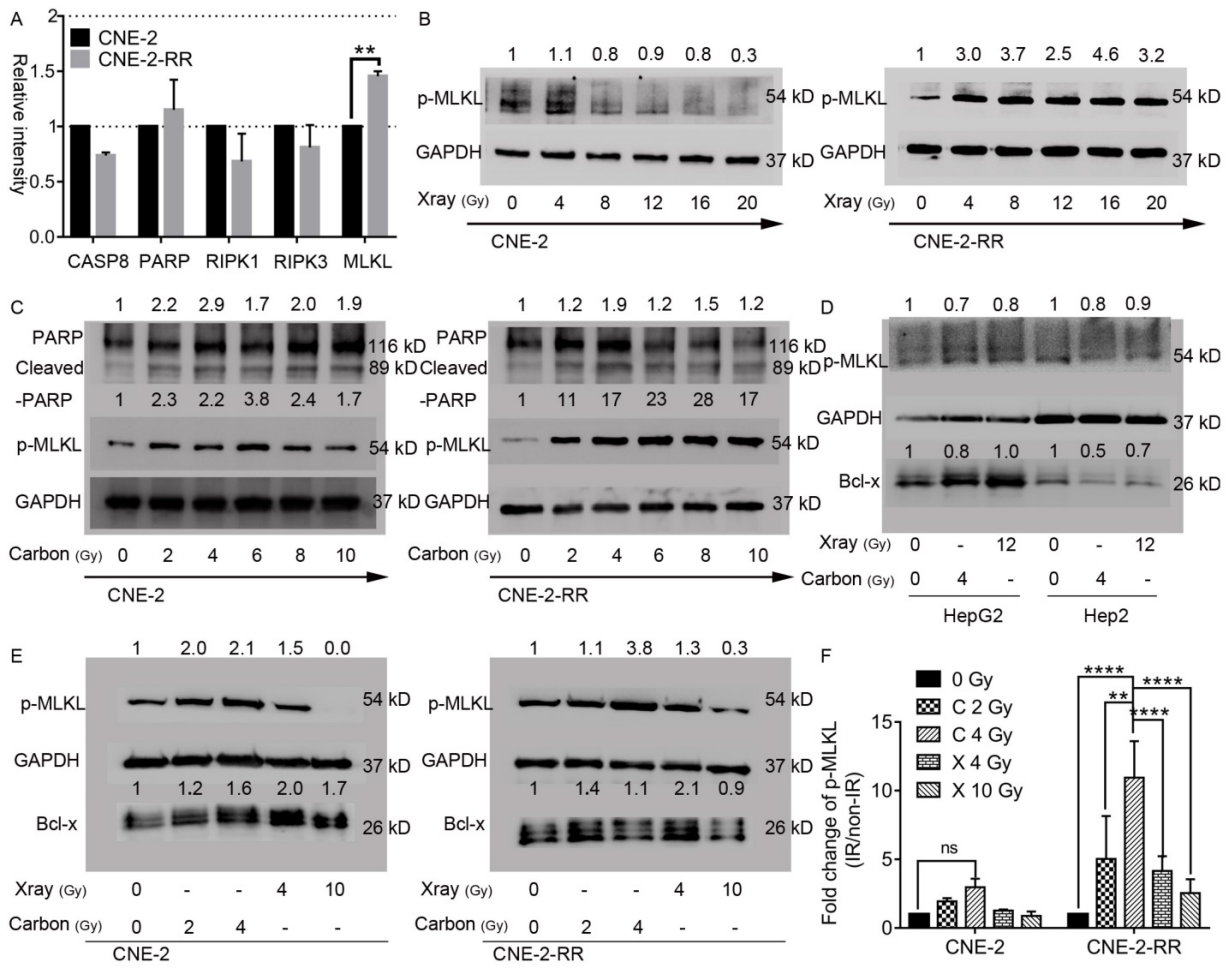
** Necroptosis induction in parental CNE-2 and photon-resistant CNE-2-RR cells following X-rays or carbon-ion irradiation.** (A) Endogenous changes of CNE-2-RR in the levels of caspase-8, PARP and other regulators of necroptosis compared with CNE-2 cells. Protein levels (mean±SD) in CNE-2-RR relative to the CNE-2 cells are presented. **P≤0.01. (B) Dose dependent phosphorylation of MLKL in X-ray irradiated cells at 48 hours. (C) Dose dependent phosphorylation of MLKL, PARP and cleaved-PARP in carbon ion irradiated cells at 48 hours. (D) Levels of phosphorylated forms MLKL and Bcl-x at 48 hours after X-ray (12 Gy) and carbon-ion (4 Gy) irradiation in HepG2 and Hep2 cells. (E) Levels of phosphorylated forms MLKL and Bcl-x at 48 hours after X-ray (4 Gy and 10 Gy) and carbon-ion (2 Gy and 4 Gy) irradiation in CNE-2 and CNE-2-RR cells. (F) Fold change in p-MLKL levels (mean ± SEM) in irradiated cells (Carbon ion: 2Gy and 4 Gy; X-ray: 4Gy and 10Gy) relative to the un-irradiated cells at 48 h. **P≤0.01, ****P≤0.0001. ns, no significance.

To further study the mechanism of carbon ion in inhibition of the photon resistant CNE-2-RR cells, endogenous levels and radiation induced changes of these regulator proteins were monitored by Western blot.** Fig. 3A** showed that endogenous levels of RIPK1 and RIPK3 were comparable in both cell lines. While 1.5-fold higher of MLKL expression of CNE-2-RR cells than that in CNE-2 cells was observed. The difference of poly-ADP-ribose-polymerase (PARP) levels between these two cell lines was not obvious, but caspase-8 was monitored to be lower in the photon resistant (CNE-2-RR) cells compared to CNE-2 cells (**Fig. 3A**). The crucial events of necroptosis include the hyperactivation of the DNA repair enzyme PARP 30 and the phosphorylation of MLKL (p-MLKL) 16. In the current study, both PARP-1 and cleaved PARP-1 was increased in NPC cells lysate after carbon ion irradiation at 48 hours (**Fig. 3C**), supporting the idea that carbon ion induced cell death is not only dependent of apoptosis, but also dependent of necroptosis. Moreover, the phosphorylation of MLKL is identified as the critical step in the induction of necroptosis resulting in its oligomerization and translocation to the cell membrane leading to the rupture of plasma membrane and promoting inflammation by the release of substantial amounts of DAMPs and cytokines 13-15, 31-33. Therefore, the p-MLKL expression following irradiation in both the cell lines after different dose of irradiation was analyzed. **Fig. 3B** and** 3C** showed that in parental cells, dose dependent downregulation (as low as 0.3 fold) of the p-MLKL level was detected after X-ray irradiation, while a significant upregulation (1.7-3.8 fold) could be seen at 48 hours after carbon irradiation. In the photon resistant CNE-2-RR cells, p-MLKL showed a 3.4 folds upregulation on average after X-ray irradiation. However, after carbon ion treatment, there was a significant 19.2 folds upregulation in average (up to 28 folds). At 48 hours after 2 Gy CIRT (**Fig. 3E, 3F and S3**), the p-MLKL level of CNE-2 and CNE-2-RR cells were 1.94±0.24 (p = 0.97) and 5.04±3.12 (p = 0.06) folds upregulation compared with control, respectively. Those values were 2.97±0.62 (p = 0.28) and 10.94±2.67 (P ≤ 0.0001) after 4 Gy CIRT. However, for NPC cells treated by X-ray, only 4 Gy (not 10 Gy) showed a trend of significantly upregulation of p-MLKL in CNE-2-RR cells 4.15±1.08 (p = 0.08). Moreover, we found that a significant upregulation of p-MLKL in the PR-NPC cells treated by carbon ion (4 Gy) compared with photons irradiation at both physical (4 Gy) and RBE (10 Gy) doses (P≤0.0001). However, both carbon ion and X-ray treatment did not upregulate the phosphorylation level of MLKL in other tumor cells (HepG2 and Hep2 cells, **Fig 3D**) at 48 hours. These results clearly showed that although X-ray could induce upregulation of p-MLKL in CNE-2-RR cells, carbon ion could induce more p-MLKL expression than X-ray for both parental and PR-NPC cells (**Fig. 3 and S3**), indicating the unique radiobiologic effects of carbon ion and could induce more necroptosis.

### Carbon ion radiation suppress necroptosis inhibitor expression in NPC cells while X-ray had the opposite tendency

**Figure 4:**
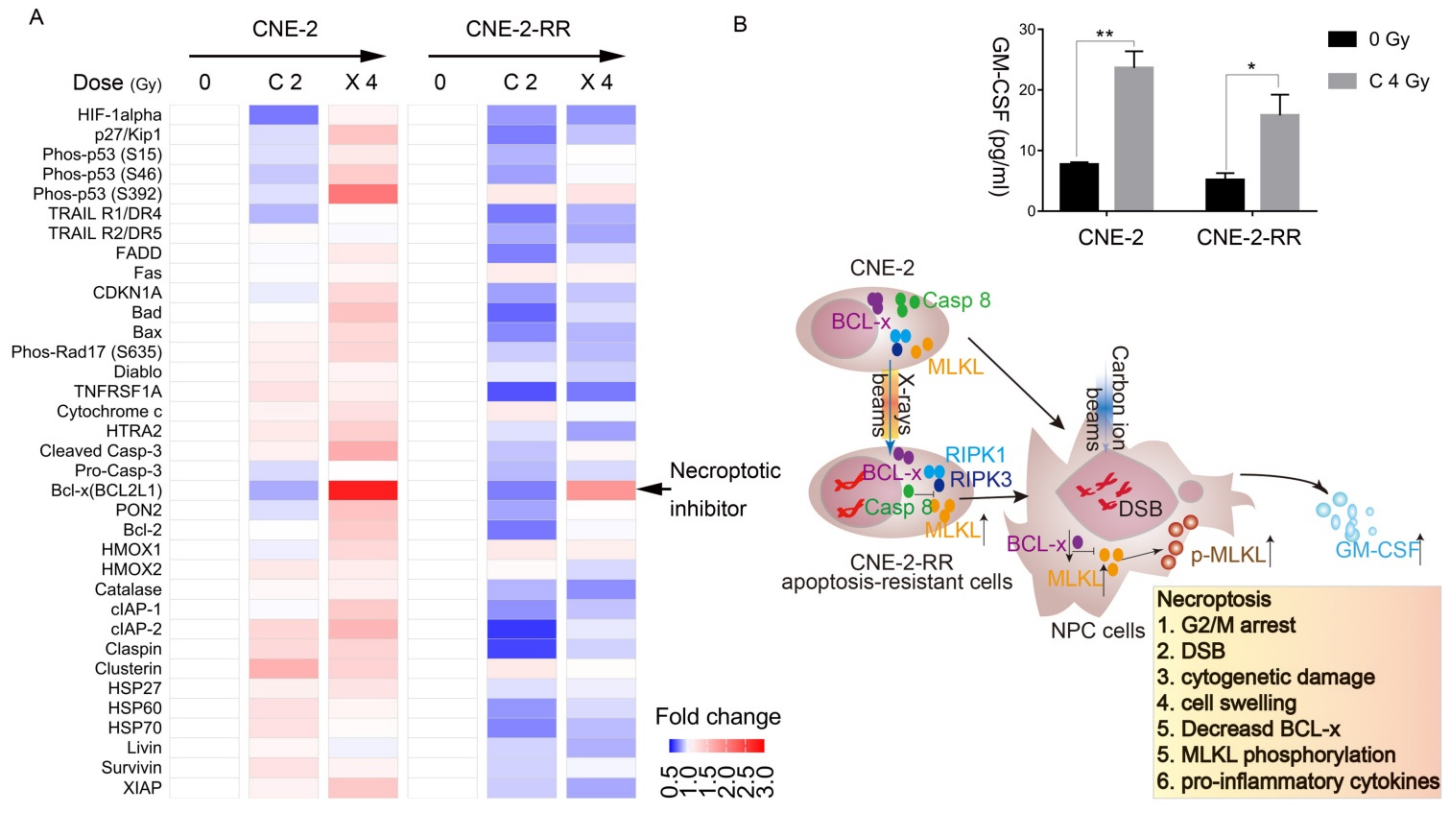
** Carbon ion radiation suppress BCL-x expression in photon resistant NPC cells.** (A) Elispot analysis of apoptotic and necroptotic regulators in CNE-2 and CNE-2-RR cells at 48 hours in irradiated cells. (B) Expression levels of GM-CSF at 48 hours after carbon ion irradiation. Values presented are mean ± SEM from 3 independent measurements. *P≤0.05, **P≤0.01. And schematic diagram of necroptotic cascade induced by carbon ion beams in NPC cells. After repeated photon irradiation, NPC cells showed photon resistance and presented relatively more predisposed to necroptosis compared to the parental cells.

The protein array next used to monitor the apoptotic and necroptotic regulator expression level after carbon ion or X-ray irradiation. The photon resistant CNE-2-RR cell derives from CNE-2 cells, but the response of these two cells to X-ray or carbon ion was significantly different. After carbon ion or X-ray irradiation, the parental CNE-2 cells tend to upregulate most of regulator, while the photon resistant CNE-2-RR had the opposite tendency. Interestingly, we found that Bcl-x was downregulated by carbon ion, but upregulated by X-ray (**Fig. 4A**), which might explain the different response of NPC cells to carbon ion and photons beams (**Fig. 3E and 3F**).

Moreover, cBioPortal analyses of the TCGA database 28 showed a significant co-occurrence (p=0.002, **Table S2**) of genomic alterations of MLKL (mainly deep deletion and mutation) and BCL2L1 (BCL-x) (mainly amplification) in pan-cancer cohorts (32 studies, 10967 patients) (**Fig. S4**). In fact, the expression of necroptotic inhibitors (Bcl-x) in CNE-2-RR cells showed lower than that in CNE-2 cells (**Fig. S5A**). We then used the STRING database 29 to demonstrate the association of MLKL and BCL2L1 (BCL-x) with other proteins and found that these two proteins are associated by direct (physical) interactions with 15 proteins, which are involved in regulating cell death pathways and inflammatory response (TNF, RIPK3, IKBKG, CASP3, CASP10, FASLG, CFLAR, FADD, TRADD, XIAP, BIRC2, CASP1, RIPK1, CASP8, HSP90AA1) (**Fig. S5B**). These results further confirmed the carbon ion effects on necroptosis induction and inflammation. Therefore, the cytokine and chemokine induced by carbon ion was further monitored by multiplexed cytokine assay. Necroptosis is typically considered a highly pro-inflammatory mode of cell death, due to release of intracellular ''danger signals'' that promote inflammation 34. Multiplexed cytokine assay (**Fig. 4B and Fig. S6**) carried out at 48 h following carbon ion irradiation, cells showed significant release of granulocyte-macrophage colony-stimulating factor (GM-CSF), which plays a critical role in development and maturation of dendritic cells and proliferation and activation of T cells, linking the innate and acquired immune response 35, 36.

## Discussion

Radiotherapy and other anticancer therapies induce multiple pathways of cell death that vary in the capability to stimulate immune responses 12, 33, 37, 38. The superiority of particle beam radiation such as carbon-ion beam with reference to their physical properties and differential dose distribution between tumor and normal tissues is well established 3, 39, 40. However, its potential to elicit a differential biological response, particularly the induction of necroptosis has not been well investigated in human tumor cells including NPC. The findings of this study raise the hypothesis that carbon ion irradiation to neoplastic cells might trigger more immune response via promoting necroptosis and therefore have important clinical implications.

In the present study, we first demonstrate that after repeated photon irradiation, NPC cells have been apoptosis-resistant cells and demonstrated photon resistance, which confirmed our results of a previous study based on another photon resistant NPC cells line 21**,** and presented relatively more predisposed to necroptosis compared to the parental cells. Cell cycle arrest has been reported as a signal of necroptosis 41. Interphase, mitotic death, mitotic catastrophe, and senescence collectively contributes to the loss of clonogenicity following irradiation. Residual DNA damage following DNA repair manifests as cytogenetic damage like micronuclei, bridges, buds. In the post-mitotic cells, which can be identified by distinctive morphological features and is linked to mitotic death that leads to delayed apoptosis and/or necrosis (or necroptosis) 24, 42. Moreover, cells undergoing necroptosis showed cell swelling morphology 32. And the necrosis (necroptosis) radio was found dose dependent after carbon ion irradiation. All the above results suggested that necroptosis may be one of the cell death pathways induced by carbon ion. Previous report showed that receptor mediated intracellular signal induced apoptosis and necroptosis are regulated by caspases, RIPK1, RIPK3 and most importantly the MLKL 13, 15, 32. And absence or reduced level/activity of caspase-8 is known to switch cellular responses to stress from apoptosis to necroptosis 43. Overexpression and knockdown of Bcl-xL, a pro-survival Bcl-2 family member, could suppress and enhanced necroptosis 44-46. Compared to parental CNE-2 cells, the lower expression of necroptotic inhibitors (caspase-8 and Bcl-x) and higher level of MLKL in photon resistant CNE-2-RR cells showed that CNE-2-RR may be relatively more predisposed to necroptosis compared to the parental (CNE-2) cells. Subsequent experiments demonstrated the significant upregulation of p-MLKL in the PR-NPC cells treated by carbon ion (4 Gy) compared with photons irradiation at both physical (4 Gy) and RBE (10 Gy) doses (P≤0.0001). Moreover, carbon ion induced a robust (up to 28 folds) p-MLKL in the PR-NPC cells as well as sensitive cells (up to 6-fold, **Fig. S3B**) coupled with a lower level of BCL-x expression and increased GM-CSF implicated in resculputure of immune system. These results suggested that carbon ion could induce necroptosis of NPC cells, especially in PR-NPC cells, and its mechanisms involve BCL-x (**Fig. 3, 4, and S4-5**).

Different types of regulated cell death induced in a context (types of therapeutic agent and cell system) dependent manner have been described so far including caspase-dependent apoptosis and various forms of caspase-independent regulated necrosis such as necroptosis, ferroptosis, pyroptosis, parthanatos, and NETosis (for review see 32, 47). In this study, carbon ion radiation induced a profound phosphorylation of MLKL (a critical event in necroptotic cell death), at the RBE equivalent carbon doses, suggesting that carbon ion irradiation preferentially stimulate necroptosis in PR-NPC cells. Necroptotic cell death is known to elicit immune responses and activate immune cells in the tumor microenvironment, which may be partly responsible for more abscopal reports 10-12. Necrostatin-1 has been found to be most effective when its delivery is delayed until 48 h after irradiation, a time that correlates with the elevation of necroptosis-inducing inflammatory cytokines and necroptosis-induced serine phosphorylation of RIPK3 in tissues 8. The delay (48 h) in the build-up of phospho-MLKL observed here (**Fig. 3**) suggests that the induction of necroptosis may be a secondary response involving paracrine signaling involving the release of DAMPS, DNA fragments and other factors initiated either by few of the highly damaged cells or by progenies of the irradiated cells. The extended G2/M block seen with both carbon ion and the RBE adjusted X-rays dose (**Fig. 2**) support to this proposition. BCL-x_L_ has been recently shown mediate RIPK3 dependent necroptosis 48. A lower level of BCL-x observed following carbon ion irradiation of PR-NPC cells (**Fig. 3E and 4A**) suggest the involvement of BCL-x in profound phosphorylation of MLKL following carbon ion irradiation of CNE-2-RR cells. IL-6 supports tumor cell survival by inducing the expression of anti-apoptotic proteins including bcl-2, BCL-x and survivin 49. Herein, carbon ion radiation promoted the release of IL-6 in NPC cells (**Fig. S6**), could further support this result. Obviously, BCL-2 family regulator plays critical role in carbon ion induced necroptosis. To our knowledge, carbon ion induced NPC cells necroptosis and the role of BCL-2 family regulator in this process has not been reported before.

The present findings have important clinical implications. First, we found that NPC cells resistant to photon (widely used in radiotherapy) are susceptible to carbon ions with a magnitude similar to the photon responsive cells (the two RBEs for 10% survival being similar). Second, carbon-ion beam induces necroptosis in both photon-sensitive and resistant NPC cells, while X-ray induces necroptosis in X-ray resistant but not sensitive cells at higher doses (**Fig. 3 and Fig. S3**). This is consistent with the recent demonstration of a lack of necroptosis induction by 4 Gy of X-ray in NPC cells 50. We also detected the p-MLKL levels of other tumor cells at 48 hours after irradiation (**Fig. 3D**), but the results showed that none of the HepG2 and Hep2 cells following carbon ion and X-ray treatment underwent necroptosis. Moreover, our results show that higher doses of carbon can induce stronger necroptosis, which is consistent with the recent finding that ablative hypofractionated photon based radiotherapy at ≥ 10 Gy per fraction induces more necroptosis than apoptosis 9. Further, a higher fraction size of 3 GyE (vs <3 GyE) or a higher biological equivalent dose significantly improved the PFS rate of patients with loco-regionally recurrent NPC after CIRT 4. Third, we also found that carbon ion induced the release of GM-CSF by NPC cells in the study, while the combination of radiotherapy with GM-CSF has been found to produces objective abscopal responses in some patients with metastatic solid tumors 51, a finding that represents a promising approach to establish an in-situ anti-tumor vaccine. Collectively, these observations suggest that carbon ion can induce necroptosis regardless of the radiosenstivity. Thus, hypofractionated CIRT may provide better survival of NPC patients via preferential stimulation of necroptosis. The enhanced sensitivity to carbon in the resistant cells derives from the induction of necroptosis, a mode of cell death and GM-CSF that can effectively elicit an inflammatory and immune response 10-12, which has the potential to enhance local tumor control as well as reduce distant metastasis, through abscopal effects, resulting in better therapeutic response. This perhaps is one of the important contributing factors for the impressive clinical responses observed in several malignancies, besides the superior physical characteristics and associated normal tissue sparing potential of carbon ions 3, 39, 40.

## Conclusion

Our study demonstrated that after repeated photon irradiation, NPC cells showed intense photon resistance and is more prone to necroptosis compared to the parental cells. Carbon ion could induce necroptosis of NPC cells, especially in photon resistant NPC cells, and its mechanisms involve BCL-2 family regulator proteins.

## Supplementary Material

Supplementary figures and tables.

## Figures and Tables

**Table 1:** Radiobiological parameters derived from the dose-response curve of CNE-2-RR cell lines.

Cell lines	Radiation	α (Gy-1) mean ± SE	β (Gy-2) mean ± SE	α/β	D_37_ (Gy)	RBE-37	D_10_ (Gy)	RBE-10	SF_2_
CNE-2-RR	X-rays	0.146±0.029	0.02±0.006	7.30	4.28	1.00	7.68	1.00	0.69
	Carbon ions	0.644±0.015	0	-	1.54	2.78	3.58	**2.15**	0.27

Note: The data was fitted to the linear quadratic model for X-ray and the purely exponential model for carbon-ion irradiation, and the parameters were determined by the fitted curve. Abbreviations: D_10_: dose for 10% survival; D_37_: dose for 37% survival; RBE: relative biological effectiveness; SF_2_: surviving fraction after 2-Gy irradiation; SE: standard error.

## Abbreviations

NPC: nasopharyngeal carcinoma
RBE: relative biologic effectiveness
PR-NPC: photon resistant nasopharyngeal carcinoma
MLKL: mixed lineage kinase domain-like pseudokinase
CIRT: carbon ion radiotherapy
LR-NPC: locoregionally recurrent nasopharyngeal carcinoma
OS: overall survival
DMFS: distant metastasis-free survival
ICD: immunogenic cell death
RIPK: receptor interacting serine/threonine kinase
DAMPs: damage associated molecular patterns
DSB: double strand breaks
PARP: poly-ADP-ribose-polymerase
GM-CSF: granulocyte-macrophage colony-stimulating factor
