# Supplementary material for: Carbon ion triggered immunogenic necroptosis of nasopharyngeal carcinoma cells involving necroptotic inhibitor BCL-x
Source: J Cancer. 2021 Jan 1;12(5):1520–30. doi: 10.7150/jca.46316 (PMC7847655; doi:10.7150/jca.46316)
Supplement: Supplementary file 1 — Supplementary figures and tables. [file jcav12p1520s1.pdf]

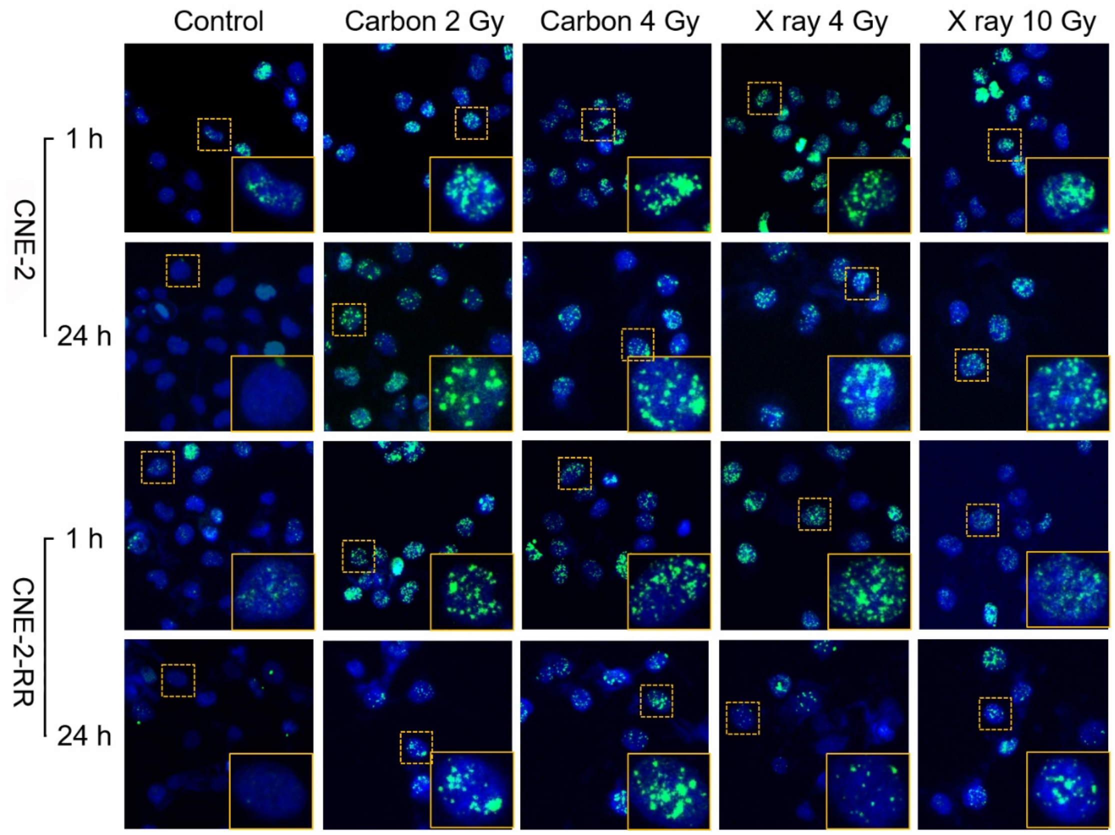

Figure S1. CNE-2 and CNE-2-RR cell DNA damage repair at 1 hours and 24 hours following X-ray or carbon-ion exposure demonstrated by fluorescence imaging of γ-H2AX loci. Original magnification: 200×.

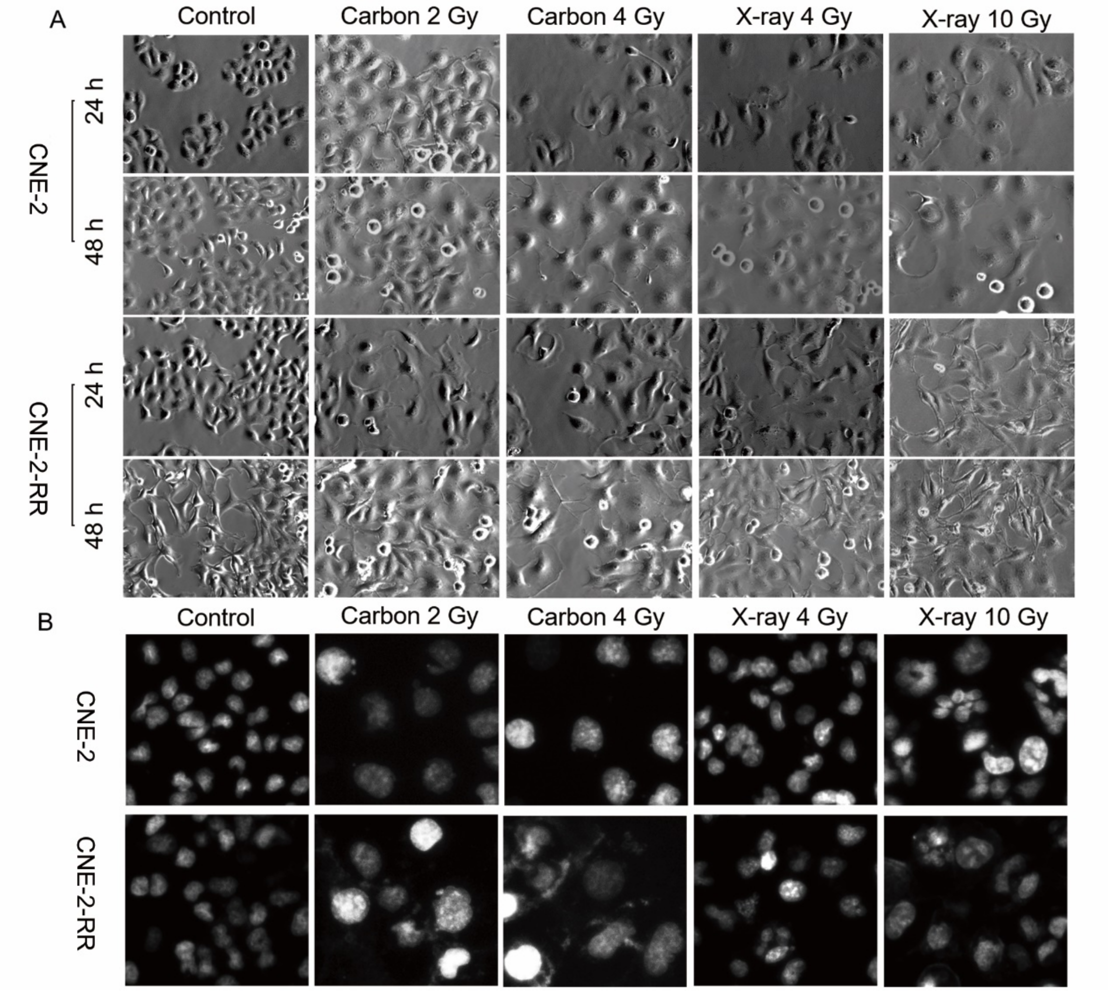

Figure S2. Cell and nuclear morphological changes of nasopharyngeal cells after irradiation and endogenous expression of necroptosis associated proteins. (A) Light microscopic images showing typical morphological features of necroptosis (flattened and enlarged cells) in carbon irradiated cells. Original magnification: 200×. (B) Fluorescence microscopy of the nuclear morphology showing a higher frequency of hypertrophy of the nucleus following carbon-ion irradiation. Original magnification: 200×.

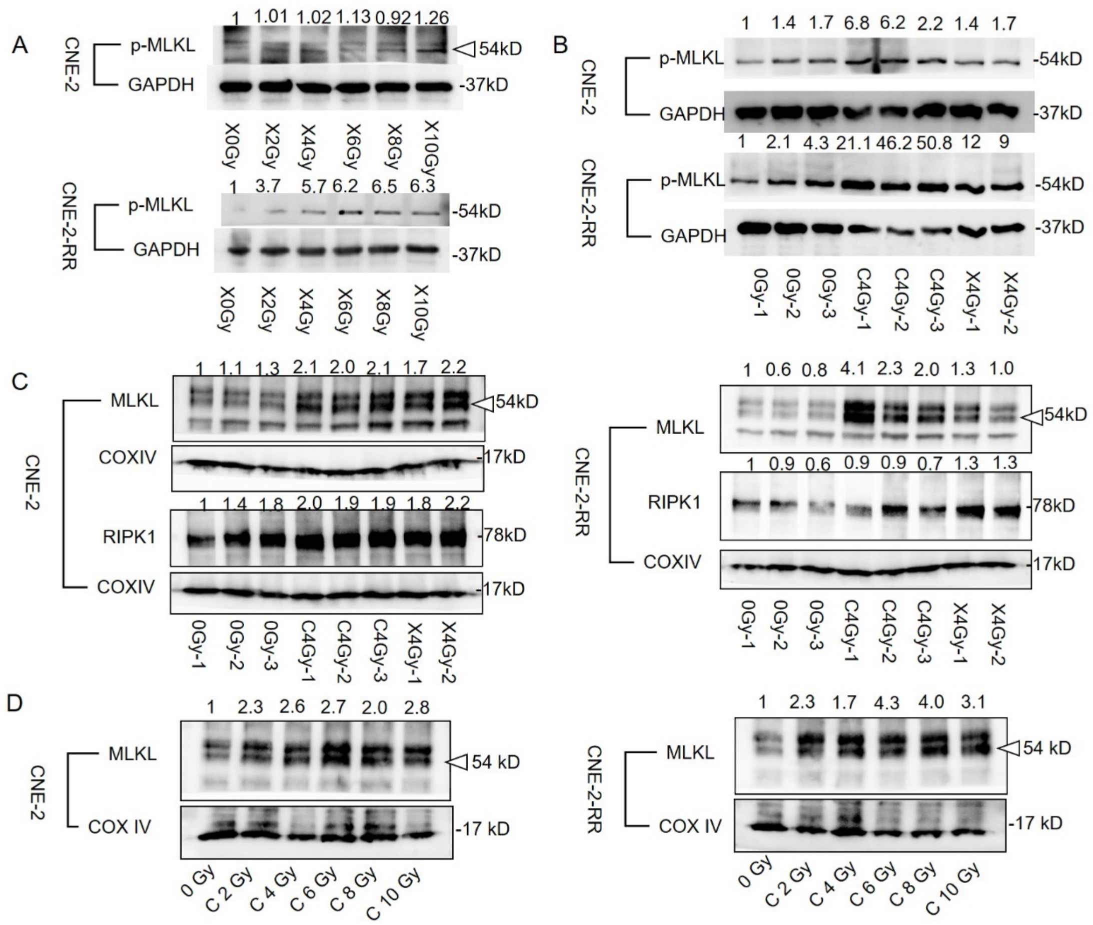

Figure S3. Radiation-induced phosphorylation of MLKL in photon-sensitive and resistant NPC cells (A) Dose dependent phosphorylation of MLKL in 0-10Gy X-ray irradiated cells at 48 hours. (B) Levels of phosphorylated forms MLKL at 48 hours after X-ray and carbon-ion irradiation in CNE-2 and CNE-2-RR cells at an absorbed dose of 4Gy. (C) Levels of MLKL and RIPK1 at 48 hours after X-ray and carbon-ion irradiation in CNE-2 and CNE-2-RR cells at an absorbed dose of 4Gy. (D) Dose dependent MLKL in carbon irradiated cells.

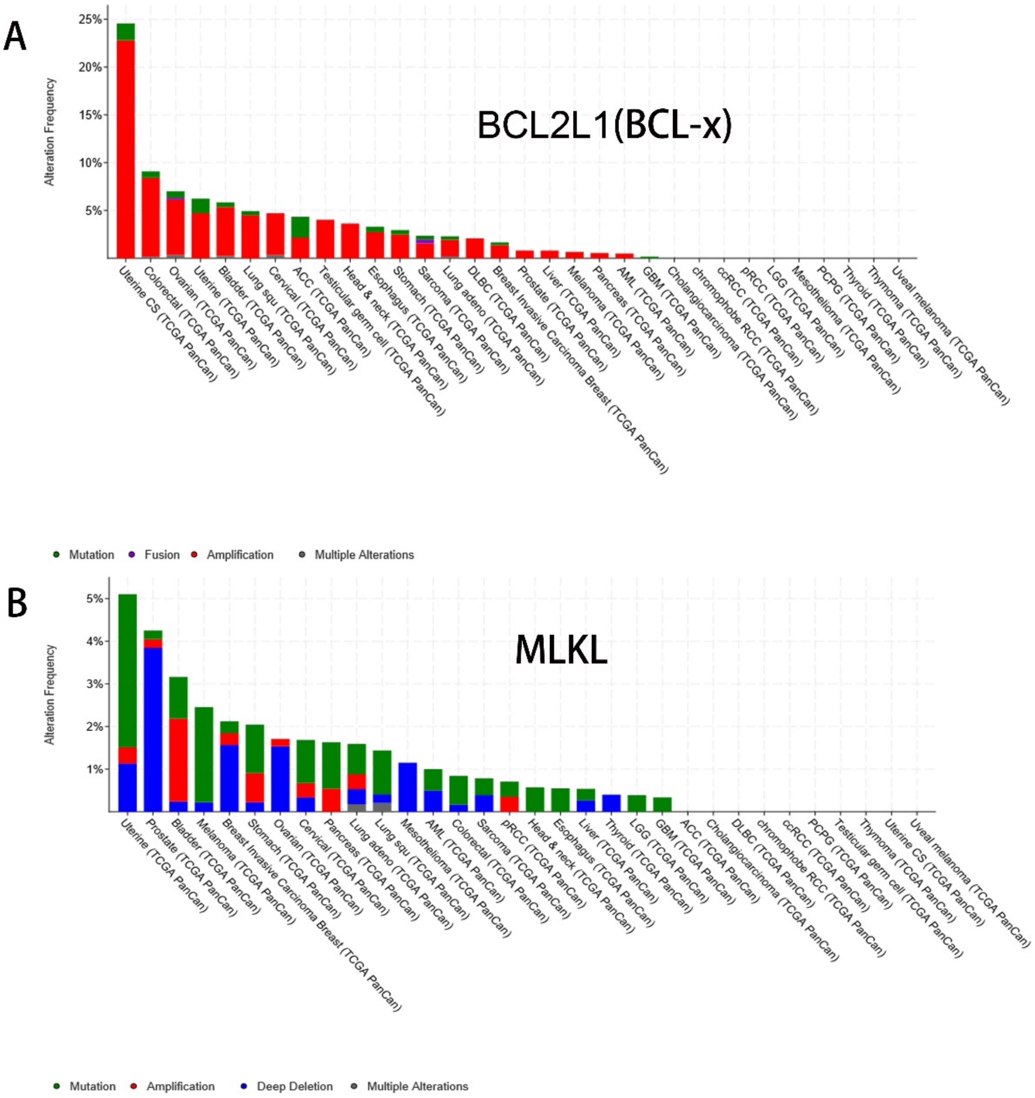

Figure S4. Genomic alterations of MLKL (mainly deep deletion and mutation) and BCL2L1 (BCL-x) (mainly amplification) in pan-cancer cohorts (32 studies, 10967 patients).

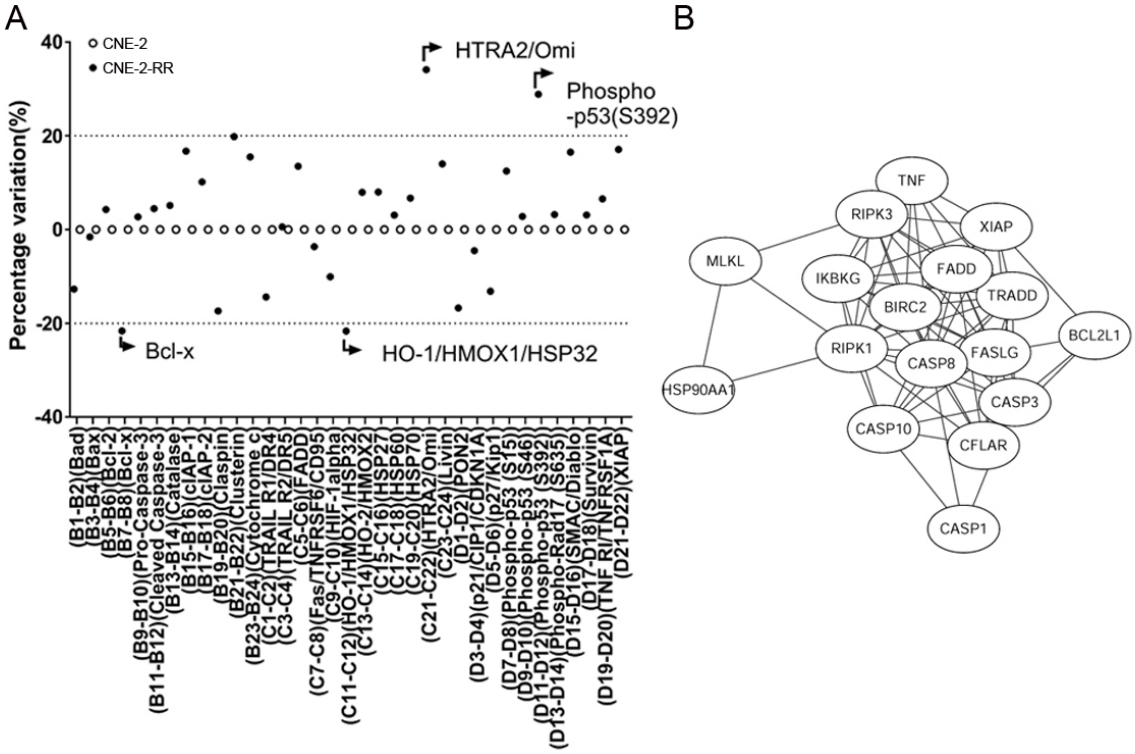

Figure S5. (A) Elispot analysis of pro- and anti-apoptotic regulators in CNE-2 and CNE-2-RR cells at 48 hours. (B) The STRING database was used to predict protein-protein association of MLKL and BCL2L1 (BCL-x), the two proteins are indirectly related by both direct (physical) interactions with 15 proteins (TNF, RIPK3, IKBKG, CASP3, CASP10, FASLG, CFLAR, FADD, TRADD, XIAP, BIRC2, CASP1, RIPK1, CASP8, HSP90AA1).

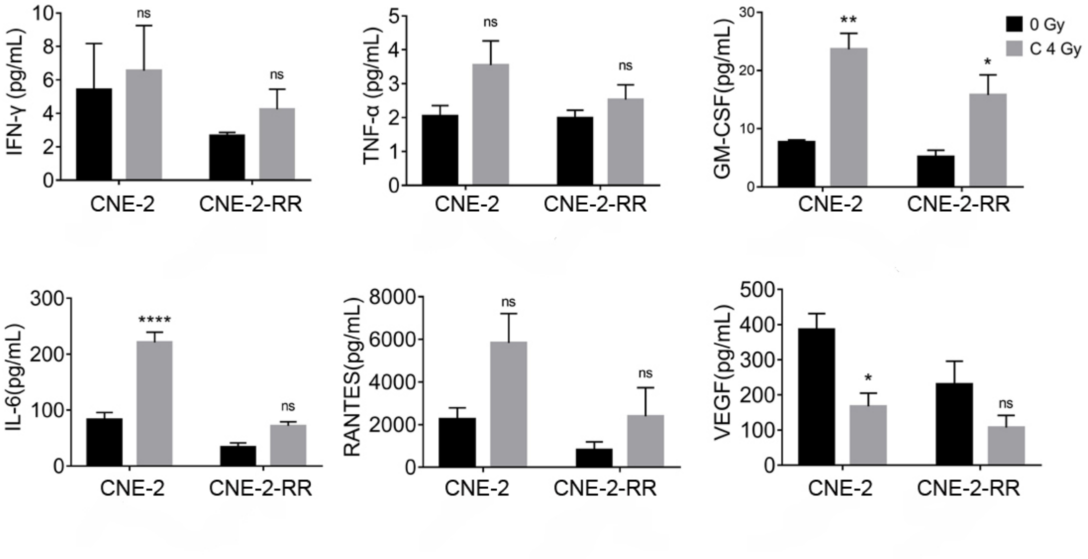

Figure S6. Levels of IFN-γ, TNF-α, GM-CSF, IL-6, RANTES and VEGF at 48 hours after 4 Gy carbon ion irradiation. Values presented are mean ± SEM from 3 independent measurements. *P≤0.05, **P≤0.01, ****p≤0.0001. ns, no significance.

Table S1 The specific 10 gene loci of STR and amelogenin genotyping in CNE-2 cell line and their radio-resistant CNE-2-RR cell line

Marker	CNE-2				CNE-2-RR			
	Allele 1	Allele 2	Allele 3	Allele 4	Allele 1	Allele 2	Allele 3	Allele 4
D5S818	11	12			11	12		
D13S317	10	12	13.3		10	12	13.3	
D7S820	10	12			10	12		

D16S539	9	10			9	10	
VWA	14	16	17	18	14	16	17
TH01	6	7	9		6	7	9
AMEL	X	X			X	X	
TPOX	8	9	12		8	9	
CSF1PO	10	11			10	11	
D21S11	27	30			27	30	

Abbreviations: X: a X-chromosome locus of the amelogenin gene.

Table S2 Co-occurrences between the indicated genes (C-BIOPORTAL/TCGA Analysis).

Gene A	Gene B	Log2 Odds Ratio	p-Value	q-Value	Tendency
BCL2L1	MLKL	1.531	0.002	0.003	Co-occurrence
